# Urinary Sodium Excretion and Dietary Sources of Sodium Intake in Chinese Postmenopausal Women with Prehypertension

**DOI:** 10.1371/journal.pone.0104018

**Published:** 2014-08-01

**Authors:** Zhao-min Liu, Suzanne C. Ho, Nelson Tang, Ruth Chan, Yu-ming Chen, Jean Woo

**Affiliations:** 1 Department of Medicine &Therapeutics, the Chinese University of Hong Kong, Hong Kong Special Administrative Region, PR, China; 2 Division of Epidemiology, The Jockey Club School of Public Health and Primary Care, the Chinese University of Hong Kong, Hong Kong Special Administrative Region, PR, China; 3 Department of Medical Statistics and Epidemiology, School of Public Health, Sun Yat-sen University, Guangzhou, PR, China; Gentofte University Hospital, Denmark

## Abstract

**Background:**

Reducing salt intake in communities is one of the most effective and affordable public health strategies to prevent hypertension, stroke and renal disease. The present study aimed to determine the sodium intake in Hong Kong Chinese postmenopausal women and identify the major food sources contributing to sodium intake and urine excretion.

**Methods:**

This was a cross-sectional study among 655 Chinese postmenopausal women with prehypertension who were screened for a randomized controlled trial. Data collection included 24 h urine collection for the measurement of sodium, potassium and creatinine, 3-day dietary records, anthropometric measures and questionnaire survey on demographic data and dietary habits.

**Results:**

The average salt intake estimated from urinary excretion was 7.8±3.2 g/d with 82.1% women above WHO recommendation of 5 g/day. Food groups as soup (21.6%), rice and noodles (13.5%), baked cereals (12.3%), salted/preserved foods (10.8%), Chinese dim sum (10.2%) and sea foods (10.1%) were the major contributors of non-discretionary salt. Discretionary salt use in cooking made a modest contribution to overall intake. Vegetable and fruit intake, age, sodium intake from salted foods, sea foods and soup were the independent determinants of urinary sodium excretion.

**Conclusions:**

Our data revealed a significant room for reduction of the sodium intake. Efforts to reduce sodium from diets in Hong Kong Chinese postmenopausal women should focus on both processed foods and discretionary salt during cooking. Sodium reduction in soup and increase in fruit intake would be potentially effective strategy for reducing sodium.

## Introduction

Reducing salt intake in communities is one of the most effective and affordable public health strategies to prevent hypertension, decrease the risk of stroke, heart and renal diseases,the three major causes of mortality globally as well aslocally [1]. Elevated blood pressure (BP) may also aggravate the positive association between urinary sodium excretion and risk of coronary heart disease [2]. Despite campaigns that encourage reduced sodium intake, excessive consumption remains a major public health problem in most populations [3].

In addition to the sodium naturally present in foods and drinking water, other important sources of sodium in our diet come from the salt used during cooking or at the table (discretionary salt), salt added in processed foods, as well as non-salt sodium containing ingredients such as monosodium glutamate (MSG) (flavor enhancer), sodium nitrate (preservative) and sodium carbonate (tenderizers) etc. Dietary survey methods tend to underestimate sodium intakes since only a portion of sodium can be captured by dietary assessment. The timed 24-hour urinary sodium excretion is considered the gold standard method to estimate intake since 85–90% of ingested sodium is excreted through the kidneys and provide an estimate of total sodium intake from all sources.

Dietary sodium intake and the major sources vary among different regions and populations, largely determined by cultural preference [3]. Awareness of contemporaneous sodium intake and identification of food sources of sodium in diets will inform more specific tailoring of salt reduction policies in local population. However, studies estimating salt consumption using timed urine samples, characterizing the food groups to assess contributions to the source of sodium as well as the associations with urinary sodium excretion are limited in Asian population. No study had been conducted among postmenopausal women with pre-hypertension or early hypertension, a population at high risk of cardiovascular diseases (CVD), declining renal function as well as osteoporosis (the major adverse medical conditions [4] [5] [6] linked with excessive sodium intake). Furthermore, potassium has been recognized as a protective factor for hypertension [7] and a proposed modifier of the association between sodium intake and CVD [8]. However, studies on the potassium intake and the contribution to urinary potassium excretion are still limited.

Thus, understanding the sodium and potassium intake among high risk populations would have enormous public health implications. The major purpose of present study was to determine the sodium intake in Hong Kong Chinese postmenopausal women with prehypertension and identify the major food groups contributing to sodium and potassium intake and urine excretion. The study will provide the basis for public health initiatives to achieve population-wide reductions in salt intake.

## Methods

The study protocol has been approved by the Ethical Research Committee of the Chinese University of Hong Kong (CRE Ref. No. 2012.201) and conducted according to the Declaration of Helsinki. Written informed consent has been obtained from all participants.

### Subjects and recruitment

This was a cross-sectional study among 726 Chinese postmenopausal women who attended a screening visit for a randomized controlled trial testing the effect of soy products supplementation on BP. Subjects were recruited from the local community by advertisement in newspapers or health talks from April to Dec, 2011. Women after prescreening interview were invited to health center for an introduction talk and training for 24 h urine collection and 3 days dietary records.

### Inclusion and exclusion criteria

Subjects were Hong Kong Chinese women aged 48–70 y; at least 1 year after the cessation of menstruation; with mean systolic BP 120∼180 mmHg or diastolic BP above 80∼100 mmHg or both. Subjects were excluded if they were on anti-hypertensive medication, hormone therapy or hypoglycemic agents in recent 3 months; medical history or presence of certain chronic diseases such as stroke, cardiac infarction, severe liver and renal diseases; breast or uterine or ovarian cancer or other malignancies in recent 5 years. More details regarding subjects recruitment were published elsewhere [9].

### Twenty-four hours urine collection

Participants were provided with a standable and graduated urine collection bag and instructed on the method of 24 h urine collection. They were required to void their bladder before starting the collection and save all urines voided from that moment onwards for the following 24 h. Participants were asked about the completeness of urine collection and to record the starting and the final collection times. The total volume of urine was measured and mixed thoroughly before aliquots. Aliquoted urine samples were immediately frozen to −85°C until analysis.

### Dietary survey, food groups and sodium intake calculation

We used 3-day food records questionnaire to estimate dietary nutrients intake. Food items were those most frequently consumed based on previous local surveys [10], [11]. The food items and reference portion size (Chinese bowl) in our dietary records were similar to our previously validated one-week food frequency questionnaire [6]. Subjects received a 30-min training on estimation of food amounts, portion and utensil sizes. Three-day food diary was completed by subjects before the visit for urine submission and checked by trained research staff. Cooked dishes and non-commercial processed foods with multiple ingredients were disaggregated into individual food items. Dietary nutrients including energy, protein, total fat, cholesterol and fiber were calculated based on the China Food Composition Table [12].

From the 3-day dietary data, the sodium consumption of the following 15 food groups was calculated: rice and noodles; Chinese dim sum; baked cereals; preserved/salted foods; dairy products; animal meat; sea foods; vegetables; soy foods; fruits; nuts; chips and cookies; beverages; soups and others. Total sodium intake was calculated by summing the estimates from all contributory food items or groups. Sodium content in each food item was adopted from Chinese Composition Table [12] and a local food sodium database [13]. We also asked participants to estimate their domestic salt consumption per month, dietary habit on preserved vegetables, fried foods, roasted meat and pickled meat, as well as their salt taste preference (in comparison with general restaurants).

### Demographic, anthropometric data and BP measurement

Individual information was collected by trained interviewers by face-to-face interview based on pretested and structured questionnaires on socio-demographic data, medical history, medication and dietary habits. Anthropometric measures such as body weight, height, waist and hip circumferences were performed according to standard protocols. BP was measured according to standard methods using a validated oscillometric technique (Omron M4-I Intellisense, Omron Corporation, Japan). Subjects were seated quietly for at least 5 minutes with the non-dominant arm supported at heart level. A cuff bladder encircling at least 80% of the arm was used to ensure accuracy. Two readings were obtained with 1 minute interval. A third BP was measured if more than 5 mmHg difference in systolic BP between two readings was noted, and the mean of the two closest was taken as the valid BP.

### Urinary biochemical testing

Twenty four hours urine samples were obtained for measurement of sodium, potassium and creatinine. The analyses were performed on Hitachi 7101 automated analyzer (Japan) at a certified clinical lab. Urine sodium and potassium were measured by ion selective electrode methods, and creatinine was measured by enzymatic colorimetric assay. The intra and inter assay coefficients of variation (CV) were 0.42% and 1.69% for urinary sodium, 0.60% and 2.01% for urinary potassium, 1.25% and 2.17% for urinary creatinine. Urinary sodium excretion was analyzed as a proxy of salt intake (g/d) calculated by the equation: 24 h urine volume (L)×urinary sodium (mmol/L)×58.4 [3].

### Statistical analysis

Analyses were performed using SPSS, version 17.0 (SPSS Inc., Chicago, IL, USA). *P*<0.05 was considered statistically significant. All variables were tested for normality using the Kolmogorov–Smirnov test and variables with skewed distribution were reported as median and quartiles. The relative contribution of food groups to the salt and potassium intake in diet were expressed as percentages. Pearson correlation and partial correlation (controlling for age, BMI and energy) analyses were performed to assess the correlations of sodium intake from various food groups with urinary sodium excretion and creatinine corrected sodium excretion (urinary sodium to creatinine ratio). Multiple linear regression analyses were used to evaluate the independent determinants of creatinine corrected urinary sodium excretion as well as urinary sodium to potassium ratio by inclusion of variables displaying significant correlations with urinary sodium by a stepwise approach. Sensitivity analyses were performed by excluding women with potentially incomplete 24 h urine collection.

The level of agreement between total dietary salt intake (salt estimated from dietary records and additional salt in home cooking) and urinary salt excretion was assessed using the Bland and Altman method [14] to identify the mean difference between the two measures.

## Results

Of the 726 women who attended the screening visit, 655 (90.2%) donated 24 h urine sample and provided 3-day dietary records, 596 (82.1%) completed anthropometric measures and questionnaire survey. Basic characteristics were indicated in **Table 1**. The mean age of the participants was 57.7±4.7 years. Of the 655 women who provided 24 h urine samples, 86 (13.1%) reported incompleteness with misses of 1–2 voids, 10 (1.5%) with total urine volume <1000 ml and 491 (74.8%) with urinary creatinine excretion in the normal range (7–18 µmol/24 h) [15]. We conducted several sensitivity analyses by excluding subjects with missed voids (n = 86) or subjects with 30% or higher coefficients of variation (n = 78) in weight corrected creatinine (Cr/Wt [16], 24 h creatinine excretion in milligrams divided by body weight in kilograms). However, there was no marked difference in the urinary salt excretion (7.6±2.9 and 7.8±3.1 g/d, respectively) in comparison with the uncorrected data (7.8±3.2 g/d).

**Table 1:** Characteristics of participants.

Characteristics	n	Mean±SD or number (%)
Age (y)	596	57.7±4.7
Age at menopause (y)	596	50.3±4.3
Years after menopause (y)	596	8.5±5.6
Body weight (kg)	596	56.2±9.0
BMI (kg/m^2^)	596	23.2±3.3
WHR	596	0.837±0.049
SBP (mmHg)	596	136.9±19.4
DBP (mmHg)	596	82.9±10.6
Hypertension (%)		268 (45.2%)
Education	596	%
Primary school and below		128 (21.6%)
Middle school		358 (60.4%)
University and above		107 (18.0%)
Occupation	596	%
Housewife		291 (50.3%)
Part-time		135 (23.3%)
Full-time		153 (26.4%)
Dietary intake	655	
Total energy (kcal/d)		2033±186
Protein (g/d)		119±102
Fat (g/d)		41.0±22.2
Cholesterol (mg/d)		259.9±132.5
Dietary vegetable intake (rice bowl)		1.5±0.8
Dietary fruit intake (small bowl)		1.6±0.8
24 h urine collection and biochemical testing	655	
24 h urine volume (ml)		2082.4±633.6
Urine Na excretion (mmol/d)		133.9±55.5
Urine K excretion (mmol/d)		57.7±19.9
Urinary sodium to potassium molar ratio		2.44±0.96
Urine Cr excretion (mmol/d)		8.5±2.7

Data are presented as mean ± standard deviation for continuous variables or number (%) for categorical variables. BMI, body mass index; WHR, waist to hip ratio; SBP, systolic blood pressure; DBP, diastolic blood pressure; Hypertension: SBP≥140 mm Hg, DBP≥90 mm Hg, or both; Na, sodium; K, potassium; Cr, creatinine. Dietary analyses were from food frequency questionnaires. 1 rice bowl of vegetable is equal to 200 g; 1 small bowl of fruit is equal to 100 g.

Dietary habit and preference were indicated in **Table 2**. Most of the women (69.5%) favored light taste diet which is less salty than foods generally provided in restaurants. The average urinary salt excretion, dietary records estimated salt intake and self-reported additional salt intake were 7.8±3.2, 5.3±2.3 and 2.2±1.9 g/d respectively (**Table 3**). According to the estimates of urinary salt excretion, 17.9% women met the WHO target of <5 g salt/d [17] and 31.6% met the China Nutrition Society target of <6 g salt/d. The urinary potassium excretion and average dietary potassium intake were 2.3±0.8 and 4.2±4.1 g/d respectively.

**Table 2:** Dietary habit and taste preference among participants.

Dietary habit and preference	Mean±SD or number (%)
Numbers of dining out per month (not including breakfast)	11.2±10.1
Salt taste preference compared with general restaurants	
More salty	4 (7%)
Similar	25 (4.3%)
A little less salty	147 (25.5%)
Less salty	341 (59.2%)
Much less salty	55 (9.5%)
Almost no salt	4 (0.7%)
Preserved vegetables intake	
≥3 times/week	21 (3.7%)
1∼2 times/week	42 (7.3%)
1∼3 times/month	144 (25.1%)
2∼11 times/yr	150 (26.2%)
<1 time/yr	216 (37.7%)
Fried foods intake	
≥3 times/week	50 (8.7%)
1∼2 times/week	169 (29.4%)
1∼3 times/month	187 (32.6%)
2∼11 times/yr	102 (17.8%)
<1 time/yr	66 (11.5%)
Roasted meat intake	
≥3 times/week	38 (6.6%)
1∼2 times/week	119 (20.8%)
1∼3 times/month	222 (38.7%)
2∼11 times/yr	124 (21.6%)
<1 time/yr	70 (12.2%)
Pickled meat/fish/sausages intake	
≥3 times/week	36 (6.3%)
1∼2 times/week	95 (16.6%)
1∼3 times/month	173 (30.2%)
2∼11 times/yr	154 (26.9%)
<1 time/yr	115 (20.1%)
Regular alcohol drinking	56 (8.5%)
Regular tea drinking	454 (80.2%)
Regular coffee drinking	208 (36.8%)

Data are presented as mean ± standard deviation for continuous variables or number (%) for categorical variables. Regular drinking means drinking alcohol, tea or coffee more than 1 time per week.

**Table 3:** Sodium and potassium intake estimated by dietary records and 24

Salt and potassium intake estimation by dietary record and 24 h urinary estimation		
	Sodium (Salt, sodium chloride)	Potassium
24 h urinary excretion		
Mean±SD (g/d)	3.1±1.3 (7.8±3.2)	2.3±0. 8
Median (g/d)	3.0 (7.5)	2.1
25th∼75th percentile (g/d)	2.2∼3.7 (5.6∼9.5)	1.7∼2.6
<5 g salt/d	17.9%	-
<5.8 g salt/d	27.8%	-
<6 g salt/d	31.6%	-
>9 g salt/d	31.1%	-
Dietary records estimated intake		
Mean±SD (g/d)	2.1±0.9 (5.3±2.3)	4.2±4.1
Median (g/d)	2.0 (5.1)	4.0
25^th^∼75^th^ percentile (g/d)	1.5∼2.5 (3.7∼6.4)	2.4∼6.1
Self-reported additional intake		
Mean±SD (g/d/person)	0.9±0.7 (2.2±1.9)	-
Median (g/d/person)	0.7 (1.7)	-
25^th^∼75^th^ percentile (g/d)	0.4±1.2 (0.9∼3.0)	-

<5 g salt/d: WHO recommendation; <5.8 g salt/d (2300 mg sodium/d): American CDC and FDA recommendation; <6 g salt/d: Chinese Nutrition Society recommendation; The conversion factor of salt from g/d to mmol/d is to multiply 17.1.

**Figure 1:**
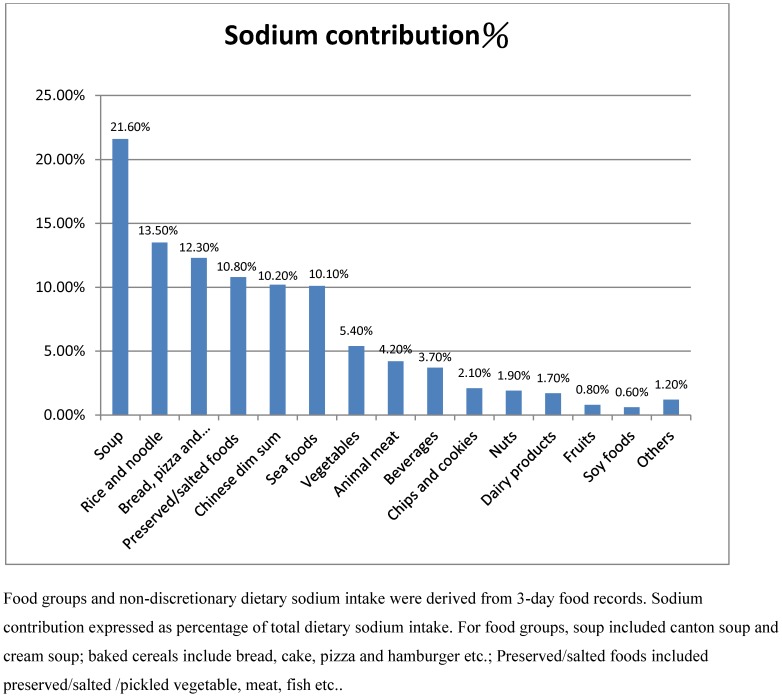
Percentage contributions of food groups to non-discretionary dietary sodium intake. Food groups and non-discretionary dietary sodium intake were derived from 3-day food records. Sodium contribution expressed as percentage of total dietary sodium intake. For food groups, soup included canton soup and cream soup; baked cereals include bread, cake, pizza and hamburger etc.; Preserved/salted foods included preserved/salted/pickled vegetable, meat, fish etc.

**Figure 1** indicated the percentiles of the food groups' contributions to dietary records estimated sodium consumption (non-discretionary salt). Of the 15 food groups, soup, rice and noodles, baked cereals, salted/preserved foods, Chinese dim sum and sea foods were the major contributors to non-discretionary salt. **Figure S1** indicated the percentiles of the food groups' contributions to dietary potassium intake. Rice and noodles, fruits and vegetables are the major contributors to the dietary potassium consumption.

Person correlation analyses (**Table 4**) indicated that creatinine corrected urinary sodium excretion was positively associated with total salt intake, sodium from Chinese dim sum, soup, preserved/salted foods and sea foods. Similar findings were observed when further controlling for age, body weight and total energy. While sodium from rice and noodles was negatively correlated with urinary sodium excretion, further adjustment for creatinine and other variables, the associations became statistically non-significant. Creatinine corrected urinary potassium excretion was also positively associated with dietary potassium intake (r = 0.09, p = 0.04) and the association remained significant after adjustment of age, BMI and total energy (r = 0.10, p = 0.03) (data not shown).

**Table 4:** Pearson correlations and partial correlations (controlling for age, weight and dietary energy) between salt intake and 24 h urinary sodium excretion.

	24 h Urinary Na excretion	Cr corrected Na excretion	Partial correlation with Cr corrected Na excretion
Salt taste preference#	−0.224**	−0.161**	−0.163**
Total salt intake†	0.112*	0.097*	0.088*
Self-estimated additional salt intake	0.064	0.050	0.061
Non-discretionary salt intake (dietary records)	0.090*	0.112*	0.116*
Dietary sodium from food groups			
Baked cereals	−0.049	−0.035*	0.054
Chinese dim sum	0.079*	0.087*	0.087*
Soup	0.026	0.083*	0.101*
Preserved/salted foods	0.113**	0.117**	0.116**
Sea foods	0.123**	0.128*	0.125*
Animal meat	0.073	0.028	0.035
Dairy products	−0.033	0.020	0.024
Chips and cookies	0.058	−0.036	−0.047
Soy foods	−0.011	−0.021	−0.050
Nuts	−0.055	−0.044	−0.042
Vegetables	−0.044	0.059	0.036
Fruits	−0.014*	0.026	0.052
Rice and noodles	−0.100*	0.010	0.014
Beverages	0.062	0.053	0.053

# The salt taste preference was in comparison with general restaurants, 1 to 5 categories corresponded to saltiest to least salty taste;

† Total salt intake was estimated from both non-discretionary salt (3 days dietary records) and discretionary salt (additional salt intake); Na, sodium; Cr, creatinine; Cr corrected Na excretion, urinary sodium to creatinine ratio;

*, p<0.05;

**, P<0.01.

Multiple linear stepwise regression analysis (**Table 5**) indicated that age, dietary vegetable intake, sodium from salted foods, soup and sea foods were positively, while total fruit intake were negatively associated with creatinine adjusted urinary salt excretion. Similar regression analysis on urinary sodium and potassium ratio (**Table S1**) indicated that BMI, sodium intake from salted foods and animal meat were positively, while dietary fruits intake, sodium intake from vegetables were negatively associated with urinary sodium and potassium ratio.

**Table 5:** Stepwise regression analysis with creatinine adjusted 24

Variables	β	SE	P value
Dietary fruit intake	−1.200	0.323	0.001
Dietary vegetable intake	0.931	0.322	0.004
Sodium intake from sea foods	1.972	0.797	0.014
Sodium intake from salted foods	1.868	0.747	0.013
Sodium intake from soup	1.077	0.439	0.014
Age	0.121	0.055	0.029

BMI, body mass index; β, standardized coefficient; SE, standard error;

Only dependent variables with P<0.05 were shown. Variables excluded include: body mass index (BMI), dietary energy (by FFQ), dietary sodium intake from bread, Chinese dim sum, animal meat, rice and noodles, and self-reported additional salt intake.

The 95% limit of agreement, defined as the mean difference between total dietary salt intake (non-discretional plus additional) and urinary salt excretion was −3.5 g (95% CI: −10.9 to 3.9 g).

## Discussion

Salt intake estimated from 24 h urinary excretion indicated that most (>80%) Hong Kong Chinese postmenopausal women had salt consumption above WHO recommendation of 5 g/d [17]. Food groups such as soup, salted foods, Chinese dim sum and sea foods were the major contributors to urinary sodium excretion. Discretionary salt intake made a modest contribution to overall intake. The study presents a comprehensive overview on salt intake in Hong Kong Chinese postmenopausal women and provides the basis for public health initiatives on the primary targets for salt reduction at a population level.

In our report, urinary salt excretion was 7.8 g/d; however if approximately 15% non-renal loss [18] were to be included, the overall salt intake would be 9.0 g/d. Studies have estimated that decreasing salt intake from 10 g to 5 g per day would reduce overall stroke rate by 23% and CVD by 17% [19]. A recent meta-analysis reported a small salt reduction of 2.0–2.3 g/day was associated with a 20% decrease in cardiovascular events [20]. The favorable effect was even observed in normotensive population [21] [22]. If sodium intake of the population can be decreased, such important health gains can be achieved with relatively small economic commitment [23]. An economic modeling study estimated that 8.5 million cardiovascular deaths could be prevented over 10 years for a 15% (1.7 g/day) reduction in salt intake achievable with a low cost of US$0.40∼1.00/person/year. With the high prevalence of hypertension in Chinese population and the heavy burden of CVD on health resources, reducing sodium intake would be an effective and affordable public health strategy.

Two international studies, INTERSALT (10079 men and women aged 20–59 y from 1985 to 1987) and INTERMAP (4680 men and women aged 40–59 years from 1996 to 1999) were by far the largest studies around the world with collection of standardized data on 24-hour urinary sodium excretion. INTERSALT [24] reported that the highest urinary sodium excretion were found in China, ranging up to 299 mmol/d (17.5 g salt/d) in men and 253 mmol/d (14.8 g salt/d) in women in the Beijing, northern China. The sodium excretion in southern China (Guangxi) was much lower with 150 mmol/d (8.8 g salt/d) in men and 128 mmol/d (7.5 g salt/d) in women. INTERMAP [25] investigated 3 counties in rural China and reported an average of sodium intake of 3.6 g/d (9.1 g salt/d) in the North and 2.5 g/d (6.35 g salt/d) in the South. Both studies reported that sodium intake in China was greatly above the international recommended intake and considerably varied by geographic regions. Our results in salt intake are in line with the data in South China. However, the two studies were conducted 20∼30 years ago and did not include samples from urban areas of China.

A number of studies have been published since then giving data on sodium intake from different regions in China. However, most of the reports were based on dietary survey methods and studies without timed urine collections. The China Health and Nutrition Survey cohort [26] which included 16,869 adults aged 20–60 y from 1991 to 2009 reported that sodium intake is decreasing but remains double the Institute of Medicine recommendations (1.5 g sodium or 3.8 g salt/d). The study reported a lower proportion of sodium from added salt (69.7% in 2009) than in other studies. The sodium intake was 4.7 g/d (11.9 g salt) in 2009 and 4.4 g/d (11.2 g salt) in South China. However, the sodium intake in this study was estimated by 3 consecutive 24-h dietary recalls and condiments.

In 1995/96, the Hong Kong Adult Dietary Survey [27] interviewed a random sample of 1010 Chinese adults aged 25∼74 and estimated the sodium intake by spot urinary excretion. Results showed that 78% of participants had a daily sodium intake of more than 2.3 g/d (5.8 g salt), and sodium intake increased with age with about 5.2 g/day (13.2 g salt/d) in men and 4.5 g/day (11.4 g salt/d) in women aged 55 and above. However, the study using spot urine to estimate sodium intake which could be less accurate than timed urine sample.

Our results in Hong Kong postmenopausal women indicated that the sodium intake in our study (7.8 g salt/d), while lower than several previous reports [26]–[28] in China, was still generally higher (>80%) than WHO recommendation [17]. The discrepancies between our results and other studies could be due to different methods for salt estimation and the different population characteristics investigated (such as region, age or health status etc.). For example, the participants in INTERMAP Chinese samples were from rural areas, younger than ours (49 vs 56) and less hypertensive patients (17%) than our subjects (45%).

In Asian countries (mainland China and Japan) [29], [30], discretionary salt use accounted for most (>70%) sodium intake and only a small proportion of sodium is intrinsic in foods. In contrast, in European and Northern American diets [31], [32], an estimated 75% of sodium intake comes from non-discretionary source such as processed or restaurant-prepared foods. Our study of source of salt intake among Hong Kong postmenopausal women showed that the pattern is intermediate between typical Asian and western diets. Our dietary survey showed that non-discretionary salt intake was 5.3 g/d accounting for 59% (5.3/9) total salt intake, thus the discretionary salt was estimated to be 41% (4.7 g/d). Since participants reported a 2.2 g/d additional salt in home cooking, the other discretionary sodium was likely from other condiments (soy sauce, MSG etc.) or non-salt sodium ingredients that we have not investigated. The relatively lower discretionary salt use in Hong Kong women compared with typical Asian diet could be due to the westernization of Hong Kong cultures, increasing consumption of western foods, processed foods and eating away from home. Thus, unlike strategies in many Asian and western countries, the efforts to reduce sodium from diets in Hong Kong population should focus on both non-discretionary salt such as processed foods and discretionary salt used during home cooking.

In our study, food groups such as soup, salted foods, Chinese dim sum, sea foods and baked cereals made major contributions to the non-discretionary sodium intake. The findings are similar to Japan [29], in which the major source of non-discretionary sodium was from commercially processed fish/seafood (15%), salted soups (15%) and preserved vegetables (13%). However, data from UK and USA [29], [33] showed that cereal and meat products accounted for the greatest proportion (38%) of household sodium intake. The discrepancies in food groups' contributions could be due to the cultural difference in dietary pattern. In western countries, bread and meat are staple foods and made substantial contribution to dietary sodium [33], while in South China, Chinese dim sum, double-stewed soup, sea foods and salted fish and egg are popular in Cantonese cuisines and are the essential contributors to salt intake. However, in the regression model, only salted foods, soup and sea foods are independent contributory factors to urinary sodium excretion. One possible reason could be that Chinese dim sum and baked cereals are significantly related with total energy intake, so that these variables become non-significant to the overall model when adjusting for energy and BMI.

In the regression model, we observed that fruit intake was negatively while vegetable intake was positively associated with urinary sodium excretion. The possible reason could be related to the colinearity of some dietary components. The Chinese style of cooking vegetables involves liberal use of salt, which would increase urinary salt excretion. At the same time fruits contains higher amount of potassium which may ameliorate the negative impact of salt intake [34]. Our study provided evidence that decrease additional salt in vegetable dishes and increase fruit intake could be effective in sodium reduction.

The observed correlation coefficient (*r* = 0.11, p<0.01) between sodium intake estimated from 3-day dietary records and urinary sodium excretion in our study was lower than INTERMAP study (r = 0.42), but is still similar to studies in Finland women [35] (r = 0.13) and African adults (r = 0.15) [36]. The relative weak correlation of dietary sodium intake and urinary excretion may be explained by measurement error of dietary assessment, the single instead of multiple 24 h urine collection, variable excretion rates, and sodium losses through other metabolic pathways such as sweat and feces etc. [37]. Error in sodium intake estimated from dietary survey may occur due to inaccurate reporting, day-to-day variation or omission of added salt or other condiments during cooking or at the table.

In contrast with the underestimation of sodium intake using urinary sodium collection, dietary estimates of potassium intake often exceeded those of urinary collection which was also suggested in our data. This is because 15∼20% of potassium is lost in the feces and another portion is excreted from sweat [37]. Compared with previous urinary data in Chinese population [38], [39], our subjects had a relatively lower urinary sodium/potassium ratio (2.4 vs 3.4 [39]) and higher potassium excretion (2.3 vs 1.9 g/24 h [38]). The relatively high potassium excretion and intake in our study might be explained by the fact that our participants were at high risk of hypertension and might be following medical advice in restricting sodium intake and increasing fruits and vegetable intake.

To our knowledge, this is the first study in Hong Kong to report findings on the major dietary sources contributing to dietary and urinary sodium and potassium. The study evaluated the characteristics of sodium and potassium intake in a population with high risk for hypertension and other chronic diseases such as CVD, declining renal function and osteoporosis. The population may benefit significantly from the reduction in sodium intake. The other strengths include the relatively large number of participants with 24 h urinary based estimates of sodium and potassium intake, high completeness of urine collection, the availability of detailed covariates to adjust for a range of potential confounders. In addition, none of the participants were on antihypertensive treatment, which may confound the relationship between intake and urinary mineral excretion and therefore reverse causation seemed unlikely.

The present study has several limitations. First, dietary survey in estimation of sodium and potassium intake is prone to a number of errors. Accurate measurement of sodium and potassium intake by dietary survey is difficult due to extensive sodium and potassium distribution in foods, and widespread use of salt or sodium compounds in food processing [31], [40]. As with 3-day food records, the included food items may not necessarily be inclusive of all the sources of sodium and potassium. The other specific sources of error in sodium include: difficulties in estimating the amount of sodium added during cooking; variation in the proportion of salt retained in food; salt loss in cooking water or left behind on the plate [31]; variation in the sodium content of manufactured foods [13]. In addition, we did not specifically investigate the consumption of other condiments except for salt, such as soy sauce, MSG etc. in cooking or at the table.

Second, urinary sodium was assessed by a single 24 h urine collection and a possible regression dilution bias may be introduced due to the daily individual variability. However, a single urine measurement is considered a more accurate measure of sodium intake at a population level [41], though possibly less accurate for individuals.

Third, our study was conducted among Chinese postmenopausal women with prehypertension. Thus, the findings may not be extrapolated to the broader populations. The participants were aware of their elevated BP and willing to register for a randomized controlled trial. This population is likely to be more health consciousness in following medical advice to reduce their dietary sodium intake [31]. However, the urinary sodium data still revealed that more than 80% women had excess salt intake above WHO recommendation. The data is likely to be higher in sodium intake among general population. In addition, our participants were mainly housewives and responsible for cooking for family. Thus, health promotion regarding salt intake and health effort targeting women of this group may be effective in community in salt reduction.

## Conclusions

Our data in Hong Kong Chinese postmenopausal women with prehypertension revealed that most women had salt consumption above the international recommendation, suggesting a significant room for reduction of the sodium intake. Efforts to reduce sodium from diets in this population should focus on both discretional salt uses during home cooking and processed foods such as Chinese dim sum, salted foods, and baked cereals etc. Sodium reduction in soup and increase in fruit intake would be potentially effective strategy for reducing sodium. Further studies are necessary in other life stage of populations to provide definitive data on salt intake, and to act as a baseline against which to monitor the impact of future salt reduction initiatives.

## Supporting Information

Figure S1 **Percentage contributions of food groups to dietary potassium intake.** Food groups and dietary potassium intake were derived from 3-day food records. Potassium contribution expressed as percentage of total dietary potassium intake. For food groups, soup included canton soup and cream soup; baked cereals include bread, cake, pizza and hamburger etc.; Preserved/salted foods included preserved/salted/pickled vegetable, meat, fish etc.
(TIFF)

Table S1 **Stepwise regression analysis with urinary Na/K ratio as dependent variable and selected variables.** BMI, body mass index; β, standardized coefficient; SE, standard error; Only dependent variables with P<0.05 were shown. Variables excluded include: age, dietary energy, sodium intake from soup, baked cereals, sea foods, Chinese dim sum, fruits, rice and noodles.
(DOCX)
