# Supplementary figures and images for: Urinary Sodium Excretion and Dietary Sources of Sodium Intake in Chinese Postmenopausal Women with Prehypertension
Source: PLoS One. 2014 Aug 1;9(8):e104018. doi: 10.1371/journal.pone.0104018 (PMC4119001; doi:10.1371/journal.pone.0104018)

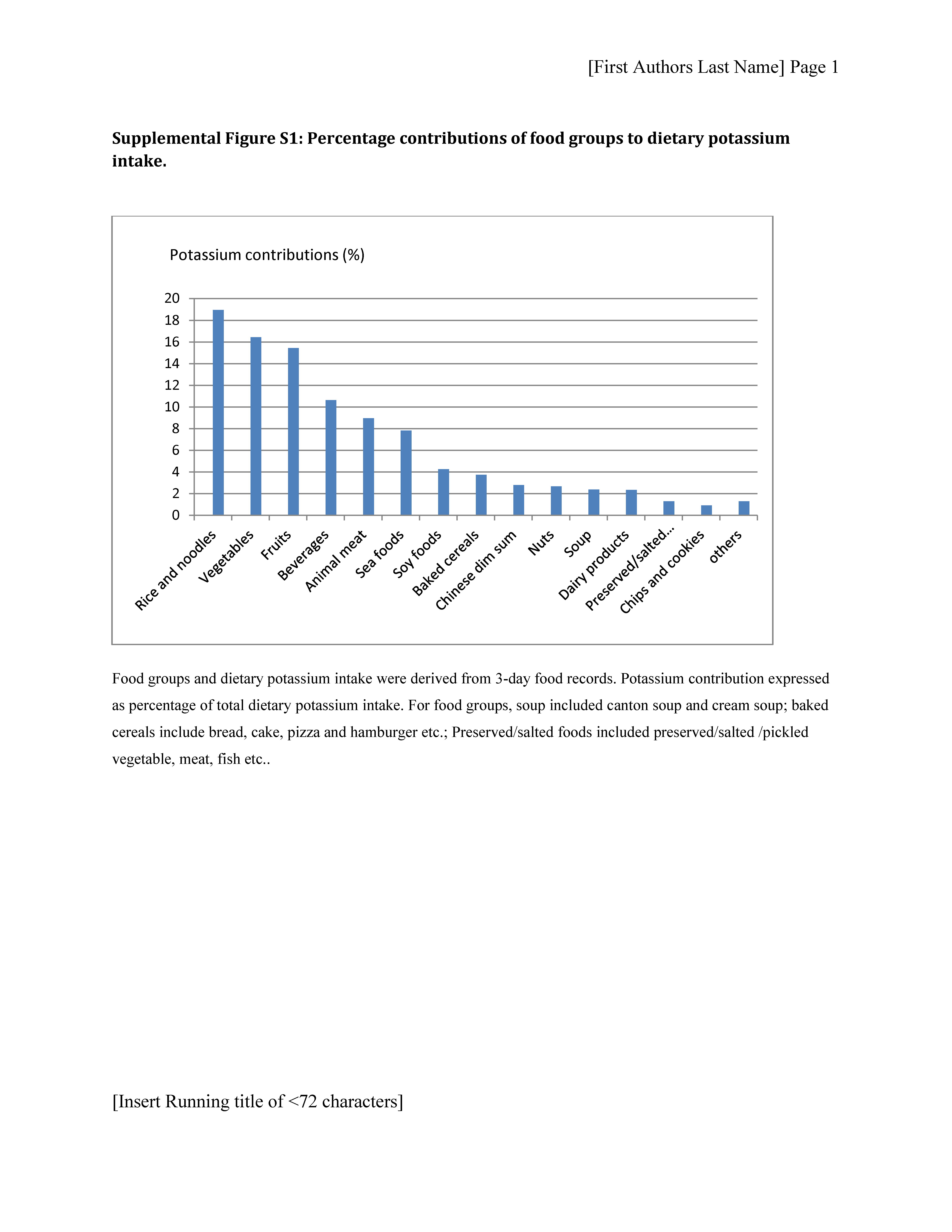

Supplement: Figure S1 — Percentage contributions of food groups to dietary potassium intake. Food groups and dietary potassium intake were derived from 3-day food records. Potassium contribution expressed as percentage of total dietary potassium intake. For food groups, soup included canton soup and cream soup; baked cereals include bread, cake, pizza and hamburger etc.; Preserved/salted foods included preserved/salted/pickled vegetable, meat, fish etc. (TIFF) [file pone.0104018.s001.tiff]
