# Supplementary material for: Urinary Sodium Excretion and Dietary Sources of Sodium Intake in Chinese Postmenopausal Women with Prehypertension
Source: PLoS One. 2014 Aug 1;9(8):e104018. doi: 10.1371/journal.pone.0104018 (PMC4119001; doi:10.1371/journal.pone.0104018)
Supplement: Table S1 — Stepwise regression analysis with urinary Na/K ratio as dependent variable and selected variables. BMI, body mass index; β, standardized coefficient; SE, standard error; Only dependent variables with P<0.05 were shown. Variables excluded include: age, dietary energy, sodium intake from soup, baked cereals, sea foods, Chinese dim sum, fruits, rice and noodles. (DOCX) [file pone.0104018.s002.docx]

**Supplemental Table S1 Stepwise regression analysis with urinary Na/K ratio as dependent variable and selected variables**

| **Variables** | **β** | **SE** | **P value** |
| --- | --- | --- | --- |
| Dietary fruit intake | -0.236 | 0.046 | 0.001 |
| BMI | 0.2 | 0.011 | 0.001 |
| Sodium intake from animal meat | 0.105 | 0.579 | 0.009 |
| Sodium intake from vegetables | -0.099 | 0 | 0.013 |
| Sodium intake from preserved or salted foods | 0.087 | 0.112 | 0.029 |

BMI, body mass index; β, standardized coefficient; SE, standard error;

Only dependent variables with P<0.05 were shown. Variables excluded include: age, dietary energy, sodium intake from soup, baked cereals, sea foods, Chinese dim sum, fruits, rice and noodles.
